# Microstructure and Properties of Ag-Doped ZnO Grown Hydrothermally on a Graphene-Coated Polyethylene Terephthalate Bilayer Flexible Substrate

**DOI:** 10.3389/fchem.2021.661127

**Published:** 2021-04-30

**Authors:** Taotao Ai, Yuanyuan Fan, Huhu Wang, Xiangyu Zou, Weiwei Bao, Zhifeng Deng, Zhongguo Zhao, Miao Li, Lingjiang Kou, Xiaoming Feng, Mei Li

**Affiliations:** ^1^National and Local Joint Engineering Laboratory for Slag Comprehensive Utilization and Environmental Technology, School of Materials Science and Engineering, Shaanxi University of Technology, Hanzhong, China; ^2^Shaanxi Province Engineering and Technology Research Center of Resource Utilization of Metallurgical Slag, School of Materials Science and Engineering, Shaanxi University of Technology, Hanzhong, China; ^3^Chengdu Hongke Electronic Technology Co., Ltd., Chengdu, China

**Keywords:** ZnO, hydrothermal, doping, flexible substrate, photocatalytic

## Abstract

Ag-doped ZnO nanorods growth on a PET-graphene substrate (Ag-ZnO/PET-GR) with different Ag-doped content were synthesized by low-temperature ion-sputtering-assisted hydrothermal synthesis method. The phase composition, morphologies of ZnO, and electrical properties were analyzed. Ag-doping affects the initially perpendicular growth of ZnO nanorods, resulting in oblique growth of ZnO nanorods becoming more obvious as the Ag-doped content increases, and the diameter of the nanorods decreasing gradually. The width of the forbidden band gap of the ZnO films decreases with increasing Ag-doped content. For the Ag-ZnO/PET-GR composite structure, the Ag-ZnO thin film with 5% Ag-doped content has the largest carrier concentration (8.1 × 10^18^ cm^−3^), the highest mobility (67 cm^2^ · V^−1^ · s^−1^), a small resistivity (0.09 Ω·cm), and impressive electrical properties.

**Graphical Abstract:**
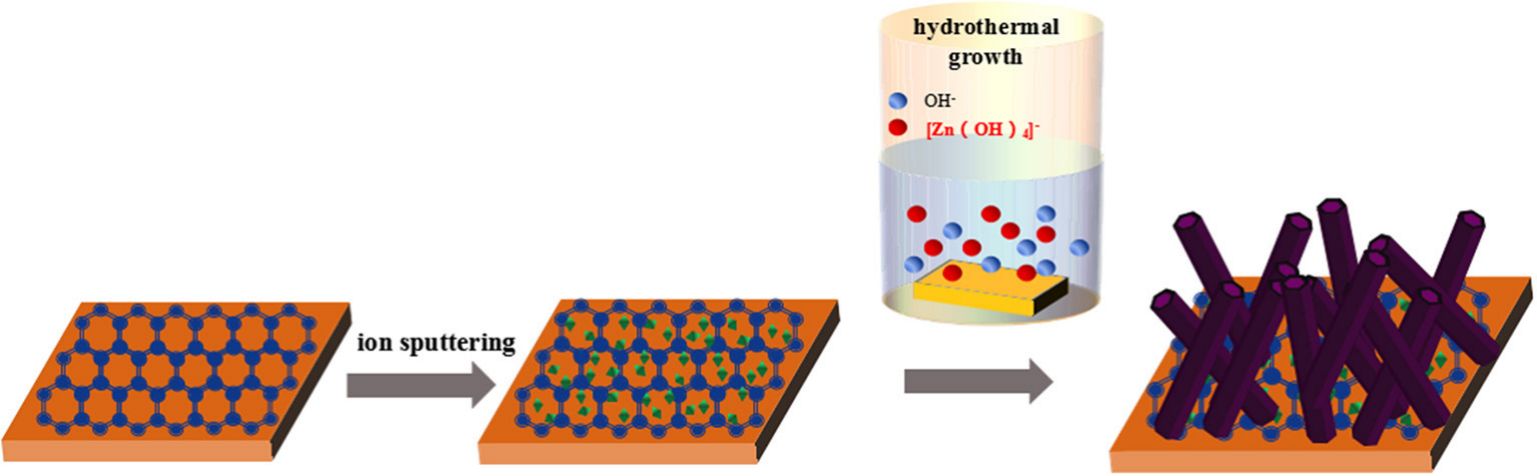


## Introduction

Flexible optoelectronic devices have changed the rigid physical form of traditional devices, greatly expanded the development and application of optoelectronic devices, and widely applied in the fields of artificial skin, batteries, sensors, intelligent robots, wearable energy collectors, flexible displays, and other high-tech fields (Lou and Shen, 2015; Chortos et al., 2016; Han et al., 2017; Li et al., 2019; Wu et al., 2019; Zou et al., 2019; Liang et al., 2020; Shen et al., 2020; Zhang P. et al., 2020; Chen et al., 2021; Zhu et al., 2021). Compared to traditional devices with silicon or germanium substrates, the flexibility of newer devices is achieved *via* a diverse range of substrates, such as conductive polymer films (Cherusseri and Kar, 2016; Guo et al., 2016; Wang et al., 2017), ultra-thin glass (Sheehan et al., 2015), graphene (GR) (Shao et al., 2015; Zhang et al., 2020; Zhang S. et al., 2020), carbon fiber cloth (Wang et al., 2015; Zhang et al., 2021), metal foil (Yeo et al., 2016), and paper-based substrates (Sahatiya and Badhulika, 2017), with polyethylene terephthalate (PET) and GR substrates are the most representative. PET offers impressive mechanical properties, high transparency, low permeability of gas and water vapor, high transmittance in the visible light range, and higher heat resistance than other polymers (e.g., polycarbonate and polyphthalamide). Graphene demonstrates peculiar electronic and physical properties, such as high tensile strength and thermal conductivity, high absorbance, large specific surface area, and quantum Hall effect at room temperature. Furthermore, the electron mobility is 140 times that of silicon (2 × 10^5^ cm^2^ · V^−1^ · s^−1^), an optical transmittance of ~97.7% (Pham et al., 2015), and the transparency and electrical conductivity are much better than those of indium tin oxide (ITO). A combined PET-GR composite flexible substrate, by adjusting the two kinds of structure unit and the intrinsic physical dimension constant, can exhibit excellent physical and chemical properties, realize synergy compensation, greatly expand the functionality of graphene-based applications (Liu and Lei, 2016), and effectively solve the mismatching of the thermal expansion coefficient between PET and ITO as well as the exfoliation phenomenon of ITO caused by device heating.

ZnO, as a photoelectric material with excellent performance, has been studied extensively by researchers all over the world in recent years (Chen et al., 2016). With the development of nanotechnology, low-dimensional ZnO nanostructures have attracted interest as the basic unit of building flexible optoelectronic devices (Chang et al., 2012; Wang et al., 2017a,b). As the smallest dimension of electron directional transmission, one-dimensional ZnO nanowires have a large specific surface area, with surface states playing an important role in regulating device performance. However, because of the intrinsic defects of ZnO, it is not suitable for direct use in devices. A common method to circumvent this limitation is to use modification treatment by element doping. Generally, n-type doping is easy to realize, there are many related studies (Wang et al., 2017a,b), and the doping technology is mature. At present, the p-type doping of ZnO has reached a bottleneck. As a group IB element, Ag is typically deployed in p-type doping. For example, Chen et al. (2011) prepared Ag/ZnO nanocomposites using a solvothermal method. The experiment found that the incorporation of Ag not only increased V_O_ in ZnO, but also caused a certain amount of Ag deposition in the composite. Therefore, owing to the degradation of methylene blue in the catalytic experiment, the photocatalytic effect of the Ag/ZnO nanocomposites was good. Moreover, Qin et al. (2008) found that doping with an appropriate amount of Ag^+^ can increase the crystallinity of ZnO and reduce the optical band gap when studying the synthesis of Ag/ZnO thin films by the sol-gel method. However, although the p-type doping of Ag is difficult owing to the self-compensation effect, the study of Ag-doped ZnO is essential. According to the calculation of the ZnO impurity level, the impurity level of Ag is close to the top of the ZnO valence band, which belongs to the shallow acceptor level (0.4 eV) (Zhang et al., 2012), so it is often used for acceptor doping of ZnO. The ion radius of Ag^+^ is much larger than that of Zn^2+^, making it easy to replace in the gap. Moreover, relative to other precious metal elements, Ag has excellent self-generation performance. Therefore, research on Ag doping is valuable for the preparation of high-quality ZnO thin films. From the perspective of materials science, the in-depth study of the properties of ZnO-based flexible films can provide a theoretical and scientific basis for the development of high-performance flexible electronic devices (Qian et al., 2016).

In this study, Ag-doped ZnO nanorods grown on the double-layer film of graphene-coated polyethylene terephthalate (PET-GR) were synthesized by low-temperature ion-sputtering-assisted hydrothermal synthesis method, by introducing a few amount of silver nitrate [Ag(NO)_3_] to zinc nitrate hexahydrate [Zn(NO_3_)_2_·6H_2_O] and hexamethylenetetramine (C_6_H_12_N_4_, HMTA), to investigate the photocatalysis mechanism of the Ag-ZnO/PET-GR composite structure. Our studies explore the controllable growth parameters of ZnO nanostructures, the relationship between process parameters and structure, and the influence of the substrate on the morphology and performance of ZnO nanostructures for the Ag-ZnO/PET-GR composite structure.

## Experimental Section

### Synthesis of Ag-ZnO/PET-GR Composite Structure

Ag-doped ZnO nanostructures grown on commercial PET-GR substrate were prepared using the hydrothermal method. Zinc nitrate hexahydrate [Zn(NO_3_)_2_·6H_2_O] of 0.5 mol/L and C_6_H_12_N_4_ of 0.5 mol/L were dissolved in deionized water to confect 30 mL of precursor aqueous solution, before silver nitrate (AgNO_3_) concentrations of 0, 1, 3, 5, 7, and 9% (where a doping concentration of 9% was defined as 0.09 mol/L) were added to the precursor solution, respectively. The reaction solution was stirred for 30 min and then transferred to a high-pressure reactor. The ZnO seed layer was sputtered onto a PET-GR substrate for 130 s using a sputtering current of 30 mA and vacuum degree of 0.1 mbar. The substrate adhered to the ZnO seed crystal was sandwiched by a specimen holder and placed vertically into an autoclave, which was completely submerged in the as-prepared mixed solution. The reactor was sealed, placed in an electric oven and heated at 90°C for 6 h. The sample was removed after cooling, and the surface-attached impurities were washed with deionized water and then dried at room temperature to obtain Ag-ZnO/PET-GR films.

### Characterization

The phase composition was analyzed by X-ray diffraction (XRD) using Cu-Kα radiation (Rigaku, D/Max 2200 PC). X-ray photoelectron spectroscopy (XPS) was used to study the elemental compositions at the surface and the chemical valence of the samples (ESCALAB 250Xi spectrometer, Al-Kα excitation source). The morphology of the samples was characterized using a scanning electron microscope (SEM, JEOL, JSM-6700F). UV-vis absorption spectra were obtained using a double beam spectrophotometer (UV-5600PC), and the adsorption equilibrium carried out in dark condition for 12 h. The electrical performance parameters of the Ag-ZnO/PET-GR composite structure were measured using a Hall effect tester (Hall-8800) at a temperature of 300 K using the Van der Pauw method.

## Results and Discussion

**Figure 1:**
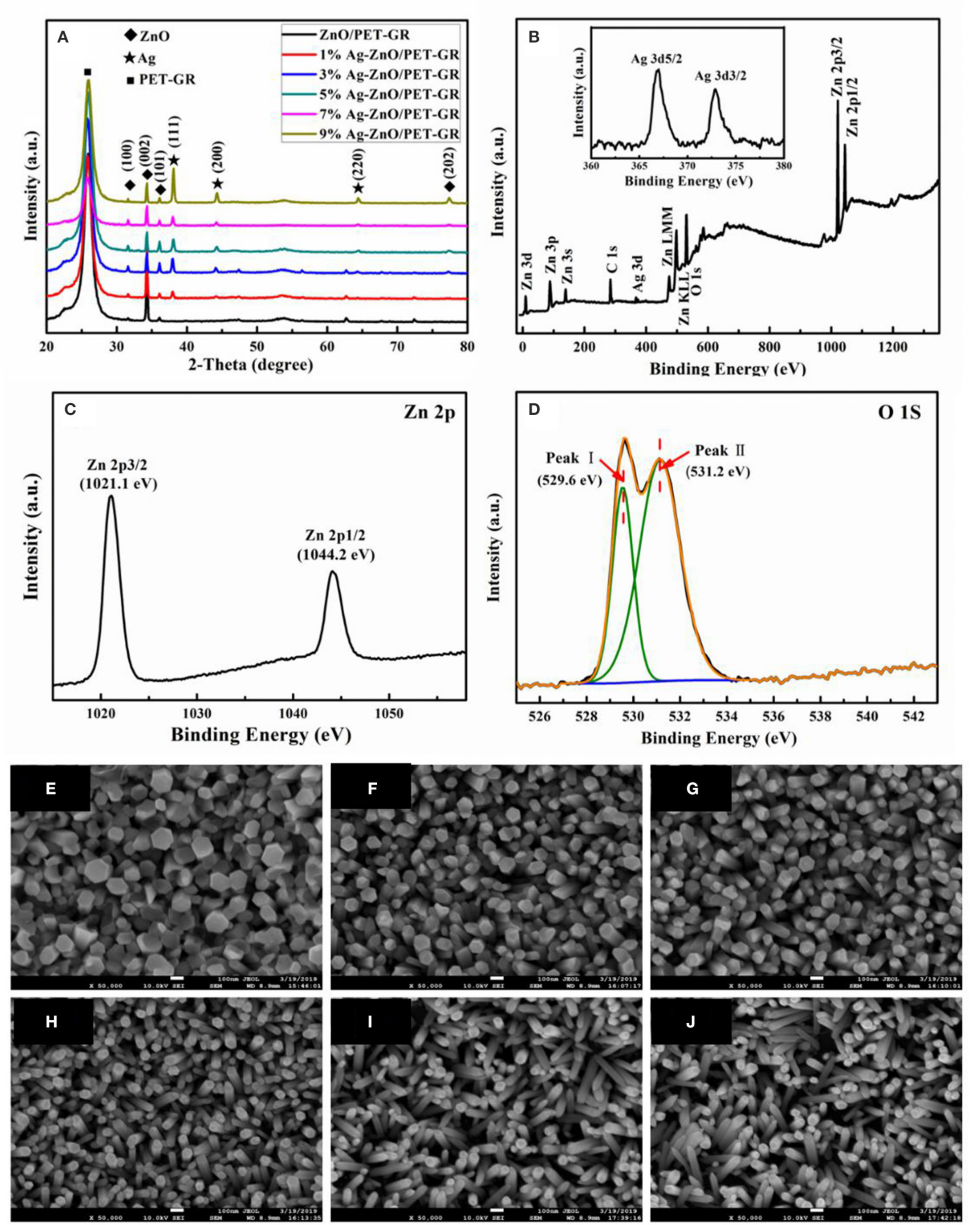
**(A)** XRD patterns of Ag-ZnO/PET-GR composite structures with different silver nitrate concentrations. The inset in **(A)** corresponds to the Ag 3d peak; **(B)** XPS spectra of the Ag-ZnO thin film with a concentration of 0.01 M; **(C,D)** high-resolution spectra at the Zn 2p and O 1s state energies, respectively; **(E–J)** FE-SEM images of Ag-doped ZnO nanostructures grown on PET-GR substrate at different silver nitrate doping concentrations: **(E)** 0%, **(F)** 1%, **(G)** 3%, **(H)** 5, **(I)** 7%, and **(J)** 9%.

Figure 1A shows the XRD patterns of Ag-ZnO/PET-GR films with different silver nitrate doping concentrations. The peaks of all the ZnO samples represented in the figure are consistent with the JCPDS card standard (No. 35-1451), and the diffraction of ZnO. The sharp diffraction peaks prove that a high-quality hexagonal wurtzite ZnO thin film is deposited on the flexible substrate. In addition to the miscellaneous peaks belonging to the PET-GR substrate in the XRD pattern, compared with the pure ZnO/PET-GR film, the Ag-doped ZnO film exhibits stronger Ag diffraction peaks, Ag(111) and Ag(200), Ag(220), which are consistent with the Ag standard diffraction spectrum (No. 36-1451) on the JCPDS database. Specifically, no silver oxide peaks appear, and the intensity of the Ag(111) diffraction peak continues to increase with increasing silver nitrate doping concentrations. In order to prove that the Ag ions have been incorporated into the ZnO lattice successfully, XPS was used to analyze the ZnO film with 1% silver nitrate doping concentration (1% Ag-ZnO). The XPS pattern of the 1% Ag-ZnO composite structure is shown in Figure 1B, with key peaks magnified in Figures 1C,D. In the full spectrum shown in Figure 1B, it can be clearly observed that the 1% Ag-ZnO composite structure contains three components: ZnO, Ag, and elemental C. The peak may be due to the adsorption of a small amount of carbon dioxide from the air onto the surface of the sample or contamination acquired during the process of C 1s detected at 290 eV. The Ag peak is weak owing to the incorporation of Ag ions, which have a small scattering cross section. It can be seen from the XPS spectrum in Figure 1B that the photoelectron binding energies of Ag 3d_5/2_ and Ag 3d_3/2_ are 367.2 and 373.2 eV, respectively. Referring to the XPS binding energy comparison table indicates that the Ag was incorporated successfully into the ZnO crystal lattice. Respectively, the two peaks at 1021.1 and 1044.2 eV in the high-resolution spectrum of the photoelectron peak of Zn 2p (Figure 1C) are attributed to Zn 2p_3/2_ and Zn 2p_1/2_. This indicates that Zn is present in the Zn^2+^ oxidation state. Furthermore, from the magnified peaks in Figure 1D, it can be seen that the O1s feature is mainly composed of two peaks at 529.6 and 531.2 eV, which correspond to lattice oxygen and surface hydroxyl oxygen, respectively. The surface hydroxyl oxygen can inhibit photo-generated electron-hole recombination and improve the catalytic activity. The crystal structure and elemental analysis of the Ag-ZnO/PET-GR composite structure reveals the existence of the Ag particles on the surface of ZnO and the success incorporated into its crystal lattice (Singh et al., 2017).

Figure 1 also shows FE-SEM photographs of Ag-ZnO thin films with different silver nitrate doping concentrations (0, 1, 3, 5, 7, and 9%) grown on PET-GR substrates. As shown in the figure, All the Ag-ZnO nanostructures exhibit nanorod-shaped structures. As the amount of silver nitrate doping concentrations increases from 0 to 9%, the diameter of the Ag-ZnO nanorods (Ag-ZnO NRs) deposited on the PET-GR substrates gradually decreases from 140 to 41 nm; in addition, the angle of the Ag-ZnO NRs relative to the substrate gradually increases, which is due to the continued increase of Ag-doped content. As more Ag nanoparticles are deposited on the sputtered ZnO seed layer, destroying the ZnO growth environment, the Ag-ZnO NRs cannot be deposited perpendicular to the PET-GR. As shown in Figure 1, as the doping concentration increases, the thickness of the ZnO nanorods in the Ag-ZnO/PET-GR composite structure becomes increasingly uniform.

The growth mechanism of Ag-ZnO/PET-GR in this experiment could be described as follows. Zn(NO_3_)_2_·6H_2_O provided the source of Zn, and (CH_2_)_6_N_4_ was decomposed to provide OH^−^, forming the growth element. The ZnO crystal is composed of many monomers with a tetrahedral structure; the geometric elements revealed at the top are the vertices of many monomers, the geometric elements revealed at the side are the edges of many monomers, and the geometric elements revealed at the bottom are the surfaces of many monomers. The addition of new monomers depends on the availability of exposed hydroxides that have not been dehydrated, so to maintain the growth of the same number of the same types of monomers, growth occurs fastest at the top, followed by the side, and slowest at the bottom, then each crystal surface of ZnO. The relationship between growth rate and magnitude is as follows: V(0001) > V(01$\bar{1}\bar{1}$) > V(01$\bar{1}$0) > V(01$\bar{1}$1) > V(000$\bar{1}$). In the experimental environment in this part of the study, the ZnO crystals grow rapidly in the (0001) direction; however, compared with the undoped ZnO film, the diameter of the ZnO nanorods in the Ag-doped ZnO films is significantly smaller. Moreover, there is no obvious hexagonal cross section, indicating that Ag doping affects the growth rate of the ZnO crystal planes.

**Figure 2:**
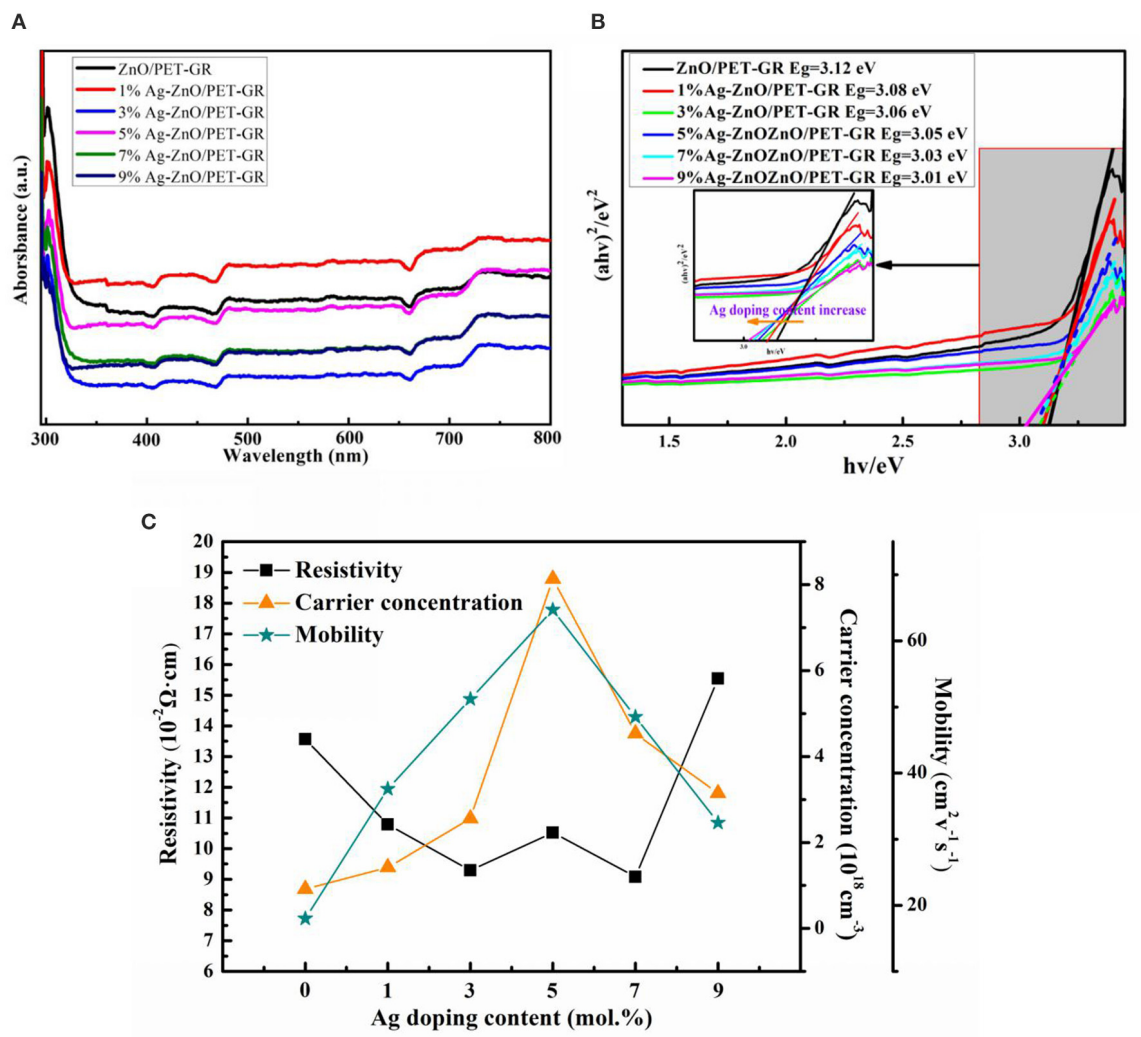
**(A)** UV-vis absorption spectra of ZnO thin films with different silver nitrate doping concentrations and **(B)** the (α*hν*)^2^ ∝ *hν* relationship diagram; **(C)** electrical properties of the Ag-ZnO/PET-GR composite structure.

In order to study the effect of silver nitrate doping concentration on the band gap of ZnO thin films, UV-vis absorption spectra of pure ZnO thin films were measured. Figure 2A shows the UV-vis light absorption spectra of ZnO films with different Ag-doping contents. From the figure, it can be seen that all samples demonstrate a certain amount of absorbance spanning the entire visible wavelength range, suggesting poor visible light transmission. This absorbance is attributed to the loading of Ag particles. For different silver nitrate doping concentrations, the ultraviolet-visible absorption capacity varies, with the absorption capacity determined by factors such as ZnO grain size and ultraviolet shielding capacity.

The UV-vis absorption spectrum assists in calculations investigating the width of the forbidden band of ZnO semiconductors. For intrinsic absorption, electrons absorb enough energy to jump directly from the valence band into the conduction band, and the intrinsic absorption must meet the energy of the photon. It must be equal to or greater than the forbidden band width E_g_ of the material, and also when the frequency (wavelength) of the photon is lower (longer) than the long-wave limit of intrinsic absorption. In addition, the optical absorption coefficient of the semiconductor decreases rapidly, which appears in the transmission spectrum as the rapid increase in transmittance indicates that the absorption edge appears in the transmission spectrum. The ZnO semiconductor forbidden band width is calculated as follows:

(1) $$\begin{array}{l} {\left(\alpha \text{h}\nu \right)}^{2}={\text{A}}^{2}\left(\text{h}\nu -{\text{E}}_{\text{g}}\right), \end{array}$$

where α is the absorption coefficient, hν represents the photon energy, E_g_ is the band gap of semiconductor materials, m = 1 for direct band gap semiconductor ZnO calculation, and A is a constant. Using the relationship based on E_g_ (Equation 1), a tangent is drawn along the steepest part of the absorption edge in order to extrapolate to (α*hν*) = 0, with the intersection with the x-axis corresponding to the forbidden band width value. As shown in Figure 2, based on the above steps, it can be seen that when the silver nitrate doping concentration is increased from 0 to 9%, the band gap of the Ag-ZnO film reduces from 3.12 to 3.01 eV. For the ternary alloy semiconductor material Ag_x_Zn_1−x_O, the band gap E_g_(x) can be expressed as E_g_(x) = (1–x)E_ZnO_ + xE_AO_-b(1–x), where A is the doping element, b is the band gap bending parameter, and E_ZnO_ and E_AO_ are the band gap energies of the ZnO and AO compounds, respectively. The addition of Ag to ZnO can change the band gap of the ZnO-based material, which can be used as the barrier and well layers of the thin film. Thus, it is crucial for ZnO-based devices. The Ag content of the Ag-ZnO film can be estimated from the relationship between the forbidden band width and the Ag concentration xAg, where E_g_(eV) = 3.12–0.0122xAg, which present a linear relationship. As the Ag content increases, the band gap of the Ag-ZnO film becomes narrower, which is consistent with the results calculated by Xu and Hou (2015) using the generalized gradient approximation plane wave pseudopotential method for the Ag-doped ZnO model.

Through the Hall effect test, it was determined that all samples are n-type conductive, and the resistivity (ρ), majority carrier concentration (n), and mobility of Ag-ZnO/PET-GR composite structure were all obtained, as shown in Figure 2C. (M) Line graph of the relationship between Ag and the amount of Ag-doping. It is observed that as the amount of Ag-doping increases from 0 to 9%, both the mobility and carrier concentration of Ag-ZnO/PET-GR increase, reaching their maximum values at a concentration of 5%, before decreasing at higher concentrations. This trend in the carrier concentration and mobility can be explained as follows: when the doping concentration is <5%, the increase in the Ag-doping concentration leads to more oxygen vacancies and holes in the lattice, thereby increasing the carrier concentration, while the Ag-doped also causes the ZnO grain boundary barrier to decrease, promoting carrier mobility. This is further evidenced by the XRD and XPS analyses.

However, after reaching a certain concentration, Ag^+^ causes a large number of defects in the ZnO lattice, thereby suppressing the carrier concentration and hindering the migration of electrons, leading to decreased mobility. As the resistivity ρ is inversely proportional to the majority carrier concentration (n) and mobility (μ), it increases with the silver nitrate doped concentration, in contrast to the carrier concentration and mobility trends, but its value is also n. The carrier concentration and mobility are largest in the 5% Ag-ZnO film, while the resistivity is slightly higher than that of the adjacent Ag-doped ZnO films (i.e., the 3 and 7% films). In the application of ZnO semiconductor films, it is expected that ZnO films have high mobility and low resistance. In the Ag-ZnO/PET-GR composite structure, the performance of Ag-doped ZnO films is better than that of undoped ZnO films. In fact, the performance is excellent, proving that Ag-doping does indeed modify the properties of ZnO films. Compared with other silver nitrate doping concentrations, the 5% Ag-ZnO film has the largest carrier concentration (8.1 × 10^18^ cm^−3^) and mobility (67 cm^2^ · V^−1^ · s^−1^) and a competitively low internal resistance (0.09 Ω·cm). Thus, the 5% Ag-ZnO/PET-GR film offers the best overall performance.

## Conclusions

In summary, Ag-doped ZnO thin films were fabricated successfully on a double-layer PET-GR flexible substrate using a simple low-temperature hydrothermal technique. ZnO nanorods were obtained. As the Ag-doping content increased, the effect of oblique growth became more obvious, and the diameter of the nanorods gradually decreased from 140–41 nm. The forbidden band width of the ZnO films decreased with increasing Ag-doping content. For the Ag-ZnO/PET-GR composite structure, the 5% Ag- doped ZnO thin film had the largest carrier concentration (8.1 × 10^18^ cm^−3^), the highest mobility (67 cm^2^ · V^−1^ · s^−1^), a small resistivity (0.09 Ω·cm), and good electrical properties.

## Data Availability Statement

The original contributions presented in the study are included in the article/supplementary material, further inquiries can be directed to the corresponding authors.

## Author Contributions

TA has put forward the proposals and supervised the whole work. YF and HW prepared materials and carried out in experiments. XZ, WB, MiL, and XF helped to characterize the performances of new composites. ZD, ZZ, LK, and MeL helped to analyze experimental data. TA accomplished the manuscript. ZD helped to check and revised. All authors contributed to the article and approved the submitted version.

## Conflict of Interest

YF was employed by company Chengdu Hongke Electronic Technology Co., Ltd. The remaining authors declare that the research was conducted in the absence of any commercial or financial relationships that could be construed as a potential conflict of interest.
